# Impact of Virtual Reality in Arterial Anatomy Detection and Surgical Planning in Patients with Unruptured Anterior Communicating Artery Aneurysms

**DOI:** 10.3390/brainsci10120963

**Published:** 2020-12-10

**Authors:** Samer Zawy Alsofy, Ioanna Sakellaropoulou, Makoto Nakamura, Christian Ewelt, Asem Salma, Marc Lewitz, Heinz Welzel Saravia, Hraq Mourad Sarkis, Thomas Fortmann, Ralf Stroop

**Affiliations:** 1 Department of Medicine, Faculty of Health, Witten/Herdecke University, 58448 Witten, Germany; ralf@stroop.de; 2 Department of Neurosurgery, St. Barbara-Hospital, Academic Hospital of Westfälische Wilhelms-University Münster, 59073 Hamm, Germany; iSakellaropoulou@barbaraklinik.de (I.S.); cewelt@barbaraklinik.de (C.E.); mlewitz@barbaraklinik.de (M.L.); hwelzel@barbaraklinik.de (H.W.S.); hsarkis@barbaraklinik.de (H.M.S.); tfortmann@barbaraklinik.de (T.F.); 3 Department of Neurosurgery, Academic Hospital Köln-Merheim, Witten/Herdecke University, 51109 Köln, Germany; NakamuraM@kliniken-koeln.de; 4 Department of Neurosurgery, St. Rita’s Neuroscience Institute, Lima, OH 45801, USA; asem.salma@gmail.com

**Keywords:** anterior-communicating artery aneurysm, microsurgical clipping, surgical planning, three-dimensional reconstruction, unruptured intracranial aneurysm, virtual reality

## Abstract

Anterior-communicating artery (ACoA) aneurysms have diverse configurations and anatomical variations. The evaluation and operative treatment of these aneurysms necessitates a perfect surgical strategy based on review of three-dimensional (3D) angioarchitecture using several radiologic imaging methods. We analyzed the influence of 3D virtual reality (VR) reconstructions versus conventional computed tomography angiography (CTA) scans on the identification of vascular anatomy and on surgical planning in patients with unruptured ACoA aneurysms. Medical files were retrospectively analyzed regarding patient- and disease-related data. Preoperative CTA scans were retrospectively reconstructed to 3D-VR images and visualized via VR software to detect the characteristics of unruptured ACoA aneurysms. A questionnaire was used to evaluate the influence of VR on the identification of aneurysm morphology and relevant arterial anatomy and on surgical strategy. Twenty-six patients were included and 520 answer sheets were evaluated. The 3D-VR modality significantly influenced detection of the aneurysm-related vascular structure (*p* = 0.0001), the recommended head positioning (*p* = 0.005), and the surgical approach (*p* = 0.001) in the planning of microsurgical clipping. Thus, reconstruction of conventional preoperative CTA scans into 3D images and the spatial presentation in VR models enabled greater understanding of the anatomy and pathology, provided realistic haptic feedback for aneurysm surgery, and influenced operation planning and strategy.

## 1. Introduction

Unruptured intracranial aneurysms (UIAs) affect approximately 3% of the adult population [1,2] and are usually diagnosed incidentally in individuals who are prescribed cerebral imaging for other reasons [3]. Development of these imaging techniques and their increased use in clinical practice has led to the increasing detection of UIAs [4]. UIAs can remain asymptomatic for many years. However, they can also cause symptoms such as by local compression of cranial nerves or rupture, leading to a life-threatening subarachnoid hemorrhage (SAH) [5].

Unruptured anterior communicating artery (ACoA) aneurysms are one of the most frequent sites of intracranial aneurysms [6] and are those most likely to rupture [7]. These aneurysms are known to have diverse configurations and complex flow conditions in the anterior circulation [8]. They are commonly treated surgically (clipping) or endovascularly (coiling) [9]. Each of these approaches is associated with particular benefits and risks [1]. Crucial factors for treatment indications and management options are especially related to aneurysm such as location, morphology including size and angulation, and the presence of a daughter sac or multiple lobes [4]. However, clipping remains an important treatment for aneurysms with broad necks, large size, intraluminal thrombus, complex branches, or previous coiling [10]. In general, the risk of aneurysm rupture needs to be weighed against the risk of treatment complications [11].

Diagnoses of UIAs, including unruptured ACoA aneurysms, and review of their therapies over time are inseparably connected with cranial tomographic imaging. The conventional radiographic studies available to delineate the size and morphologic features of an intracranial aneurysm are computed tomography angiography (CTA), magnetic resonance angiography (MRA), and digital subtraction angiography (DSA), which is considered the gold standard [12]. These modalities result in two-dimensional (2D) images. The ability to generate three-dimensional (3D) images from 2D images might improve the radiographic evaluation, and thus facilitate decisions regarding the appropriate treatment strategy [13].

The 3D reconstructions of imaging modalities, which were originally mainly presented on flat screens, enabled better understanding of spatial and anatomical relationships. In the 1990s, several articles on surgical virtual reality (VR) were published [14]. In recent years, VR technology has become increasingly important in many medical fields, including neurosurgery [15,16]. The current VR visualization technology enables transition from conventional 3D screen images to interactive 3D-VR models. This is associated with many benefits for operation planning, explanation of surgical procedures for patients, medical deduction and for clinical training, such as improved understanding of the detailed anatomy and configuration of cerebral aneurysms [17,18,19,20]. The continuous advances in medical technology and development of portable electronic devices has improved the user friendliness of VR technology for operators, medical students, nursing staff, and other employees integrated into the healthcare system [18].

The concept behind modern VR is the transformation of 3D images into the stereoscopic patient model, using computed tomography (CT) and MRI scans to create an exact and accurate representation of the complex anatomy using a cost-effective method, with additional implementation possibility in procedures such as minimally-invasive and endoscopic surgery [21,22]. Furthermore, preoperative 3D-VR models have been reported to be in high agreement with intraoperative conditions; the resulting intraoperative “déjà vu” feeling strengthened surgical confidence [23].

In our study, we retrospectively evaluate a cohort of patients who underwent surgical treatment for unruptured ACoA aneurysms. We intend to answer the question whether 3D-VR-based visualization of reconstructed preoperative CTAs would result in a recommended surgical strategy that deviated from the recommended strategy based on conventional interpretation of the same, orthogonal-orientated screen CTA scans.

## 2. Materials and Methods

The study protocol was approved by the ethics commission of the Medical Faculty, Witten/Herdecke University (Ref-Nr. 201/2018).

### 2.1. Patient Enrolment

In analyzing our hospital information system, we retrospectively identified patients within a 6-year period (2014–2019) who underwent surgery for an aneurysm, and matched the following inclusion criteria: (1) adult age, (2) unruptured ACoA aneurysms, (3) asymptomatic patients, (4) preoperative reconstructable, thin-slice cranial CT and CTA (1 mm slice thickness) with axial, sagittal, and coronal views, (5) microscope-integrated fluorescent videoangiography, (6) surgical procedure with clipping. To obtain a homogeneous patient group, the following exclusion criteria were defined: (1) young age, (2) multimorbid patients, (3) other cranial pathologies (tumor, angiome, etc.), (4) previous craniotomies, (5) ruptured aneurysms with SAH, (6) aneurysm therapy with coiling, (7) previous clipping or coiling, (8) recurrent aneurysms, (9) multiple aneurysms.

### 2.2. Data Acquisition and Handling

Data from all included patients were retrospectively analyzed. Patient data were collected from patient files, discharge papers, surgical reports, outpatient reports, and imaging reviews. Patient- and disease-related data, including age, gender, preoperative imaging, aneurysm morphology, aneurysm direction, perioperative complications (during surgery and within the first two weeks after surgery), and discharge-status, were collected, analyzed, and evaluated.

### 2.3. Neurosurgical Technique

The surgical procedure was performed according to a largely uniform technique, as follows. The head was fixed in a three-pin Mayfield headholder after suitable positioning. An incision according to the planned approach (pterional [24], extended pterional [25], supraorbital subfrontal [26]) was performed. The scalp and galea flap were mobilized and reflected inferiorly and/or laterally. The bone flap was removed with the help of a cranial drill and a craniotome. Under microscope, the dura mater was then opened and the brain gently retracted to locate and prepare the aneurysm. A titanium clip was then placed across the neck of the aneurysm. Intraoperative indocyanine green (ICG) videoangiography (Flow 800) was used to check clipping sufficiency [27,28]. The dura mater was then closed, and the bone flap was reinserted into the cranial defect and fixed to the bone with miniplates and miniscrews.

### 2.4. Virtual Reality Visualization Technique

**Figure 1:**
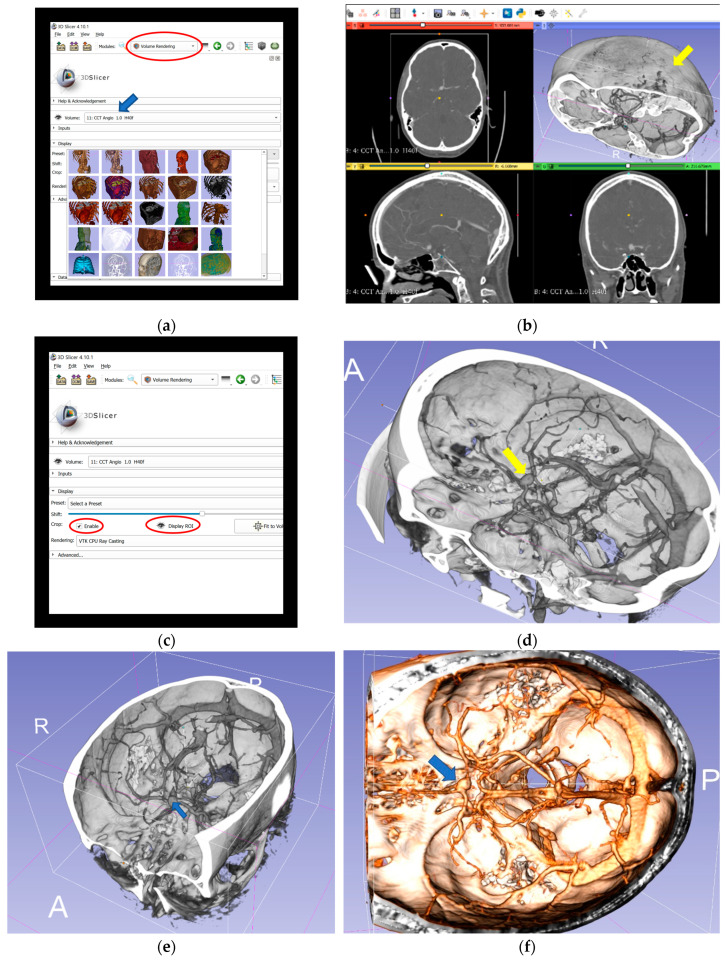
Reconstruction process of 3D-VR images and completion of the final VR scene in 3D Slicer. (**a**) Import of the original CTA data in anonymized DICOM format into 3D Slicer software to create a patient-specific database and selection of “CCT Angio-Default” (blue arrow) in volume rendering window (red circled). (**b**) Performance of 3D-VR reconstruction of skull and vessels (yellow arrow) in volume rendering window. (**c**) Activation of ROI function (red circled), which enables visualization of the aneurysm and relevant vascular anatomy through skull bones from different perspectives. (**d**) Lateral aspect of the aneurysm (yellow arrow) and relevant vascular anatomy, simplified using ROI function. (**e**) Oblique lateral aspect of the aneurysm (blue arrow). (**f**) Superior aspect of the aneurysm (blue arrow) with different color display. 3D, three-dimensional; CCT, cranial computed tomography; CTA, computed tomography angiography; DICOM, digital imaging and communications in medicine; ROI, regions of interest; VR, virtual reality.

The digital imaging and communications in medicine (DICOM) files of preoperative CTA scans were retrospectively reconstructed to 3D-VR images. We used open-source medical image analysis and visualization software (3D Slicer, Surgical Planning Laboratory, Harvard University, USA) [29], which runs on a VR workstation (main board: Intel Core i7-6800 K (Intel Corporation, Santa Clara, CA, USA); RAM: 16 GB; graphic card: 2 × NVIDIA GTX 1080 (NVIDIA Corporation, Santa Clara, CA, USA)) connected to the HTC Vive (HTC Corporation, Xindian District, New Taipei City, Taiwan) goggles, and the SteamVR tracking and controller system (Valve Corporation, Bellevue, WA, USA). The steps of reconstruction process are shown in Figure 1.

### 2.5. Study Design

**Figure 2:**
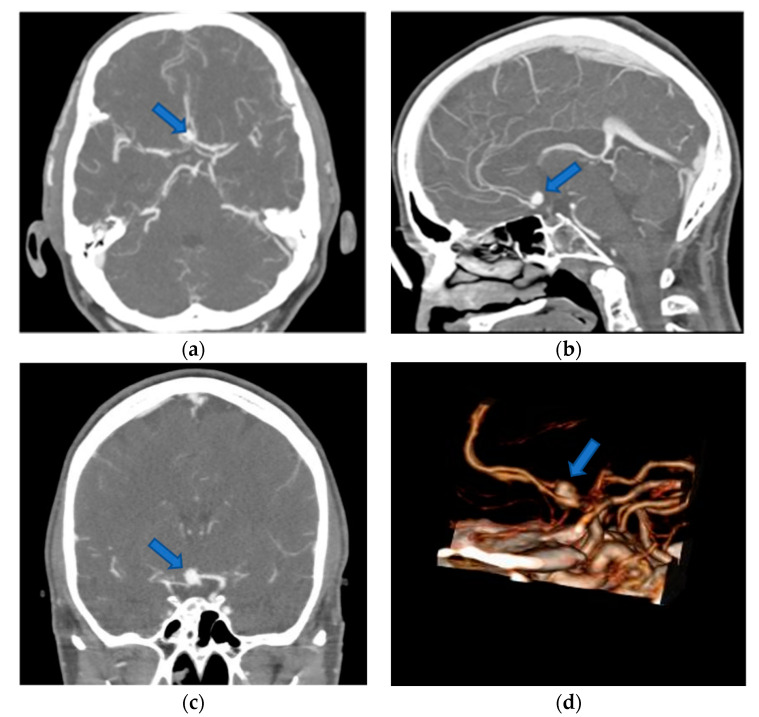
Preoperative 2D-CTA and screen 3D-CTA images of patients with unruptured ACoA aneurysm (blue arrows). (**a**) Axial 2D-CTA image presenting anteriorly directed ACoA aneurysm; (**b**) sagittal 2D-CTA image presenting superiorly directed ACoA aneurysm; (**c**) coronal 2D-CTA image presenting superiorly directed ACoA aneurysm; (**d**) lateral screen 3D-CTA image presenting anteriorly directed ACoA aneurysm. 2D, two-dimensional; 3D, three-dimensional; ACoA, anterior communicating artery; CTA, computed tomography angiography.

**Figure 3:**
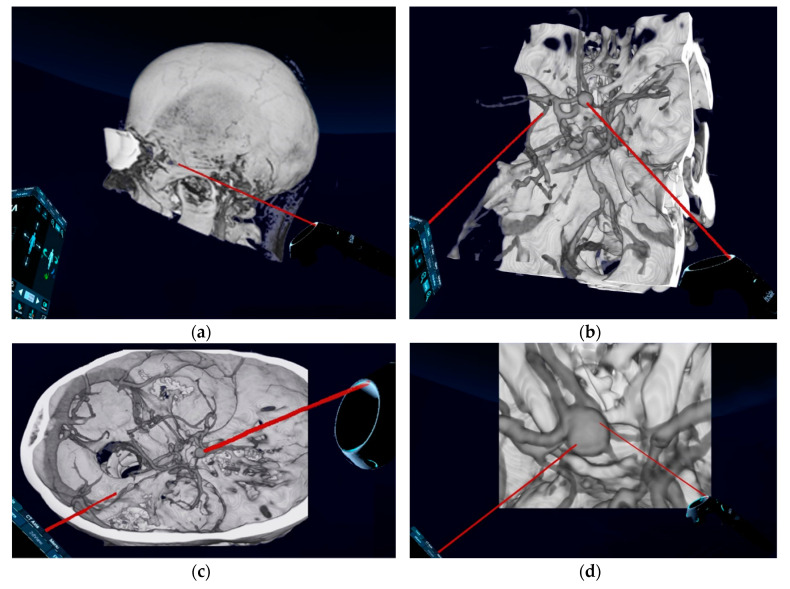
Preoperative reconstructed 3D-virtual reality images of patients with unruptured ACoA aneurysm. (**a**) Lateral aspect of the skull; (**b**) oblique superior aspect of the aneurysm and relevant vascular anatomy and the skull base; (**c**) lateral superior aspect of the aneurysm and relevant vascular anatomy and the skull base; (**d**) zoomed superior aspect of aneurysm and relevant vascular anatomy. 3D, three-dimensional.

Conventional preoperative screen CTA scans (examples in Figure 2) of all included patients were retrospectively demonstrated to ten experienced, board-certified neurosurgeons who have at least 10-years-experience in vascular neurosurgery with at least 50 aneurysm clippings performed by each of them (to reduce any influence on the recommendations given and to avoid bias, neurosurgeons who performed the operations and who have done the reconstructions were excluded). They were asked to evaluate the identification of anatomical structures, as well as determine the preferred patient and head positioning, the surgical approach and approach side, and the clipping strategy using a questionnaire (Table 1). The reconstructed 3D-VR images (examples in Figure 3) of the same patients were retrospectively presented to the same neurosurgeons four weeks later, but in a different order to minimize the influence from the first questionnaire on the second. Again, the neurosurgeons were asked via the same questionnaire. To avoid influence from the patient- and disease-related data on the image evaluations, these data were not presented. The possible influence of the preoperative reconstructed 3D-VR images compared to the conventional preoperative CTA scans (2D and screen 3D) on detection of anatomical structures and on surgical planning and strategy was evaluated.

Since the questionnaire was retrospective, the questioned neurosurgeons could give answers regarding the preferred surgical strategy that were different to the procedures that were actually carried out in these patients. To verify the confidence of the questionnaire, the intrarater reliability was tested, as each of the ten neurosurgeons could suggest different evaluations for the same image set by repeated questionnaires.

### 2.6. Statistical Analysis

Patient data were collected anonymously. We applied the Fisher-exact test [30] to estimate the statistical probability of a correlation between two variables by measuring the difference between the collected data and expected values, which would be assumed for uncorrelated factors. We assumed a *p*-value < 0.05 to be significant. For age and morphological parameters, mean ± standard deviation (SD) values were calculated. The intrarater reliability was determined using Cohen’s kappa coefficient [31].

## 3. Results

### 3.1. Patient- and Disease-Related Data

In analyzing our clinic database, 112 patients were treated for unruptured ACoA aneurysms within a 6-year period (2014–2019). The endovascular therapy was carried out in 53 (47%) patients and the surgical therapy with clipping in 59 (53%) patients. Twenty-six patients met the inclusion criteria. All had asymptomatic unruptured ACoA aneurysms and were operated with clipping. The mean age was 54 ± 7 (range 30–74) years. The aneurysm was <11 mm in 77% of patients and Anteriorly directed in 46%. Further patient- and disease-related data are summarized in Table 2.

### 3.2. Role of Image Presentation Modality in the Identification of Anatomical Structures and Surgical Planning

Questioning of ten neurosurgeons to evaluate the 26 patients, first displaying conventional screen CTA images, then presenting reconstructed 3D-VR images, resulted 260 reply sheets each (520 total). For the determination of the intrarater reliability, substantial agreement was found (kappa values = 0.67 to 0.79).

#### 3.2.1. Impact on Identification of Anatomical Structures

The 3D-VR modality showed a significant advantage in the visualization of the aneurysm, as well as the surrounding arterial anatomy compared to the conventional CTA; 57% of questioned neurosurgeons found the 3D-VR-based anatomical depiction to be appropriate, compared to 40% of neurosurgeons viewing the CTA images (*p* = 0.0001) (Table 3). As expected, fewer (45%) neurosurgeons who viewed the 3D-VR models required a DSA to better assess the aneurysm and arterial anatomy, compared to 57% of neurosurgeons viewing the conventional CTA images, which showed significant difference in favor of 3D-VR (*p* = 0.008) (Table 3).

#### 3.2.2. Impact on Selection of Patient and Head Positioning

The supine position was mostly recommended by the neurosurgeons, independent of image presentation technique (80% using CTA, 87% using 3D-VR). Thus, the visualization technique showed no influence on the recommended patient positioning (supine/other positions; *p* = 0.38) (Table 4). However, the recommended head positioning was significantly influenced by the image visualization modality (straight or “neutral”/ straight with flexion/straight with extension/lateral rotation; *p* = 0.005), with the lateral rotation being mostly selected (71% using CTA, 81% using 3D-VR) (Table 4).

#### 3.2.3. Impact on Selection of Surgical Approach and Approach Side

The pterional approach was mostly recommended using the CTA image presentation method (36%), while the extended pterional approach using 3D-VR (50%). The image presentation technique had a significant influence on the selected surgical approach (supraorbital subfrontal/pterional/extended pterional; *p* = 0.001) (Table 5), but no significant influence on the recommended approach side (right/left; *p* = 0.25) (Table 5).

#### 3.2.4. Impact on Selection of Clipping Strategy

With both image presentation methods, neurosurgeons mostly did not recommend temporary clipping (72% using CTA, 76% using 3D-VR). Thus, the decision to use a temporary clip or not as part of the aneurysm clipping strategy was not significantly influenced by the visualization technique (*p* = 0.32) (Table 6). Similarly, the straight/curved clip was mostly chosen by the neurosurgeons for the permanent clipping (67% using CTA, 71% using 3D-VR); again, the image presentation technique showed no influence on the selected permanent clip type (straight/curved/angled/fenestrated; *p* = 0.54) (Table 6).

## 4. Discussion

In our retrospective study to evaluate the impact of the image visualization modality on surgical planning in patients with unruptured ACoA aneurysm, the way in which sectional images were viewed (i.e., conventional or 3D-VR) significantly influenced the identification of aneurysm-related anatomical structures and an important part of the recommended surgical strategy. The neurosurgeons interviewed evaluated the images retrospectively, without prior knowledge of the surgical procedures that were performed on the patients. The patients with ruptured and symptomatic aneurysms were excluded, since their surgical strategy is not only selected on image-based vascular anatomy presentation, but also other key factors such as the neurological condition and the presence and severity of intracerebral or subarachnoid hemorrhage.

Evaluation of clinical results: The literature data differ significantly with regard to perioperative morbidity and mortality rates after surgical clipping. Bekelis et al. reported in a retrospective cohort study on mortality rate of 0.7%, unfavorable discharge of 15.3%, stroke of 5.3%, hydrocephalus of 1.5%, cardiac complications of 1.3%, and deep vein thrombosis of 0.6% [32]. The overall morbidity and mortality rates were relatively low in a study by Moroi et al. (0.0% and 0.6%, respectively) [33]. However, these rates were higher in a meta-analysis by Kotowski et al. (1.7% and 6.7%, respectively) [34] and in a study by Ogilvy et al. (15.9% and 0.8%, respectively) [35]. Despite the differences in study size, demographic data, and aneurysm morphology, the clinical results in our study, with morbidity rate of 4% and mortality rate of 0%, are generally in comparable range of the literature data including studies mentioned here.

Role of 3D-VR models in the detection of ACoA aneurysm-related anatomical structures: ACoA aneurysms are considered to be complex due to their multiple vascular relationships, deep location, and frequent anatomical variations [6,36]. Microsurgical clipping of these aneurysms necessitates perfect surgical strategy, based on review of the 3D angioarchitecture and abnormalities of the patient’s ACoA complex with its ACoA aneurysm [6,37], using several radiologic imaging methods [12].

DSA is the gold standard for diagnosis and anatomical evaluation of cerebral aneurysms. 3D-DSA reconstructions allow accurate assessment of aneurysm morphology and accurate demonstration of the anatomic relationship between the aneurysm and the ACoA complex [38]. However, DSA is a costly and time-consuming invasive examination, with a risk of complications [39]. Furthermore, in unilateral angiography only the ipsilateral A1 and A2 segments, without association with the bony skull base, are usually clearly demonstrated [38]. This makes noninvasive methods for detection of aneurysm-related anatomy and planning of the therapeutic procedures more attractive.

CTA is a low-cost, noninvasive, and rapidly-acquired imaging modality, with a lower risk of neurologic complications [40]. CTA has been shown to adequately predict the ipsilateral and contralateral anatomy around the ACoA aneurysm [41]. Although 3D-CTA reconstructions of preoperative 2D images are now well established to simplify the vascular anatomical presentation of ACoA aneurysms, they do not completely approximate the anatomy realized under the operating microscope at surgery [38]. Additionally, they are mainly presented on flat screens, which are of different and sometimes insufficient sizes and qualities. Therefore, it is useful to integrate an image presentation modality such as VR, which combines the advantages of the other modalities into one system, with fewer undesired characteristics.

Reportedly, VR systems generate clear and illustrative virtual 3D images that clearly show the location, size, and shape of the aneurysms. They provide precise imaging details similar to screen 3D-DSA, but additionally give the possibility of exact anatomical spatial relationships of the aneurysm to the parent arteries and to the skull [42]. In other studies, VR visualization technology provided a close resemblance to the real surgical anatomy and enhanced the surgeons’ spatial understanding of the individual vascular anatomy [43]. Accordingly, compared to conventional screen CTA (2D and screen 3D) in our study, the 3D-VR modality showed a significant advantage in visualizing the aneurysm as well as the surrounding arterial anatomy, and thus significantly reduced the need for DSA among the neurosurgeons interviewed (Table 3). An explanation for these results could be that the VR-based observation of the same image modalities allowed a completely free perspective of the anatomical structures from all directions. The surgeon can “step” into the images and gain different insights into the anatomy and explore the different structures, while having the feeling to be part of the VR environment [44]. According to our experience, this technique provides a much more intuitive understanding of the present situs, and even more of the underlying pathology. The VR technique shows the anatomy with a higher magnification and more detail than common radiologic images. VR-based visualization helps to improve radiological evaluation, since limiting factors such as suboptimal background illumination, reflective glare, and visual disturbances can be eliminated, and the object can be focused in front of the goggles [18].

Role of 3D-VR models in the selection of patient positioning and surgical approach: The aim of microneurosurgical management of ACoA aneurysms is total occlusion of the aneurysm sac with preservation of flow in all branching and perforating arteries [45]. To achieve this objective, it is important to consider aneurysm-related factors such as neck width and shape, wall calcifications, and branching vessels, as well as approach-related factors such as patient positioning, and location and extent of craniotomy [13]. The optimal head placement, can optimize exposure of important vascular and neural structures, provide less brain retraction, and lead to safer surgeries through suitable approaches [46]. The pterional approach became the standard for treatment or exposure of AComA aneurysms. However, other skull base approaches are also widely used [36]. The key factors that guide the selection of surgical approach type and side include consideration of aneurysm morphology, aneurysm projection, A1 dominance, and relationship of the aneurysm projection to A1 dominance and to the plane of the both A2 vessels [36,47].

For planning of patient and head positioning and surgical approach, the evaluation of conventional preoperative imaging is essential. Despite many advantages including the complete visualization of all tissues around the aneurysm, which could influence the choice of surgical access strategy, conventional imaging methods have limitations regarding the spatial representation [38]. Using VR technique and by selection of certain default modes in the reconstruction process, such as “CT Soft-Tissue-Default” instead of “CCT Angio-Default” (Figure 1a), a complete view of tissues surrounding the aneurysm is also possible. However, the quality of the arterial representation in these modes, based on currently available computational processing system and algorithms, is sometimes not appropriate. Additionally, the spatial navigation along the vessels and surgical corridor is significantly restricted by the reconstructed tissues. Therefore, we have focused on the representation of the skull and vessels, using “CCT Angio-Default” mode, as an important factor for the surgical planning and tested VR technology as an alternative for planning the surgical approach and strategy. In the literature, the choice of head positioning and surgical approach was reported to be significantly influenced by the VR visualization technique [43,48]. This is in accordance with the findings in our study, where the extended pterional approach and thus the lateral head rotation was mostly selected (Table 4 and Table 5). An explanation for this result could be that the view on conventional CTA images does not correspond to the direction of the view on the operative site. The neurosurgeons need to look at 2D-CTA and screen 3D images to create mental spatial 3D reconstructions of the aneurysm, aneurysm-related arterial anatomy, and skull. This process is often difficult and stressful and differs greatly among neurosurgeons. Moreover, due to limitations of mental reconstruction abilities, information might be lost or mentally not precisely processed [44]. The transformation of conventional preoperative screen CTA images into 3D-VR images, through specific software and technical equipment, simplifies this process. Moreover, the 3D-VR models can facilitate not only the spatial reconstruction of the aneurysm, but also its relationship to skull base and bony structures, as well as to other superficial anatomical landmarks. The neurosurgeons can freely rotate and position the patient and the head in virtual space. They can also enlarge the head and the vascular structures to the maximum size and navigate along the appropriate corridor and along the vessels and from one structure to another. VR provides realistic haptic feedback for aneurysm surgery [43]. These possibilities that VR technology enables are limited in normal screen 3D reconstructions; therefore 3D-VR image presentation play an important role in the choice of the head position and surgical approach.

With regard to patient positioning, our study showed that retrospective selection was not influenced by the method of viewing the preoperative images (CTA or 3D-VR). ACoA aneurysm clipping is mostly carried out using a supraorbital subfrontal, pterional, or extended pterional approach. This means that the majority of these aneurysms can be well approached with the patient in the supine position, without the need for more complicated and time-consuming lateral or other positionings. This may explain the higher (albeit non-significant) choice of the supine position, independent of image presentation modality (Table 4). Similarly, the image visualization technique did not influence the recommended approach side, with a tendency toward the right-sided approach using both modalities (Table 5). The factors that determine the approach side, in particular A1 dominance and aneurysm projection, seemed not to need and thus were not influenced by the spatial presentation and could be well identified in conventional CTA as in 3D-VR images. For this reason, the results showed no deviations.

Role of 3D-VR models in the selection of clipping strategy: Prior to clipping, adequate dissection and exposure of the entire “H” complex, separation of the aneurysm neck or dome from the perforators, and preservation of the parent vessel are the key to a successful outcome. Therefore, the selection of the clip shape and size that matches the configuration of the ACoA complex and preserves the blood flow to the perforators is important to reach these objectives [13,36]. Moreover, in complex and large aneurysms, it is also important to know whether protection using temporary clips is necessary [6,49].

The planning of the clipping strategy was influenced by the VR visualization technique in studies of ruptured and unruptured aneurysms of different locations, sizes, directions, and forms [13,48,50]. However, in our study the VR presentation technique showed no influence on the selected clipping strategy. Independent of the image presentation modality, the neurosurgeons mostly recommended not to use a temporary clip and to use a straight/curved clip (Table 6). The explanation for this difference could be that the unruptured ACoA aneurysms presented in our study were mostly smaller than 11 mm and directed inferiorly or anteriorly. Compared to other large, complex, or ruptured aneurysms included in other studies, these aneurysms probably could be accessed without temporary clipping. In addition, the configuration of the ACoA complex could mostly be restored with a straight/curved clip. The role of these factors (size and direction) in clip selection seems more important than the image presentation modality, which may explain our results concerning clipping strategy.

Study limitations and further prospects: VR plays an increasing role in many scientific neurosurgical studies, but does not yet represent a routine application. The VR technique can support improved orientation toward anatomical relations of aneurysms, but may also tempt surgeons to neglect the complexity of approaches that leads to a different access strategy. Moreover, the anatomy of small branches and perforators and the evaluation of adhesions and its severity is still missing in both 3D-CTA and VR. Further innovative technologies, such as the reproducing of physical 3D cerebral aneurysms models, provide very precisely replicated patient-specific anatomy [51]. These technologies can be used together with VR technology for training and teaching and provide important preoperative information for treatment strategy. To what extent the 3D-VR image data presentation can lead not only to a change in the surgical strategy, but also to a favorable change in surgical complication rates or patient outcomes, can only be answered by a prospective, multicenter study. VR is still dependent on the quality of input data. At the same time, however, with the possible clinical applications of 3D-VR technology and its consecutive relevance for the surgical strategy, technical and procedural quality requirements and standards must also be defined for the technical equipment and software algorithms. This could improve the quality of VR visualization technology, clarify the real borders between 3D-CTA reconstructions and VR, and better demonstrate VR promising features in comparison to other modalities.

## 5. Conclusions

In our retrospective study in patients with an unruptured ACoA aneurysm, the reconstruction of conventional screen preoperative CTA scans into 3D images to enable spatial presentation in VR models did not influence existing and established patient positioning methods and often-used clip forms. However, the VR technique allowed better detection of aneurysm-related anatomical structures and significantly influenced the selection of head positioning and surgical approaches and thus an important part of the surgical planning and strategy.

## Figures and Tables

**Table 1:** Questionnaire regarding the anatomical structure detection as well as the recommended surgical strategy for patients with anterior communicating artery aneurysms, using conventional computed tomography angiography (CTA) images and three-dimensional virtual reality presentations.

Surgeon’s Name:
Patient-ID:
1. How is the identification of anatomical structures according to the presented images? □ Appropriate □ Not appropriate
2. Is a preoperative cerebral digital subtraction angiography (DSA) necessary? □ Yes □ No
3. Which type of patient position would you choose for the surgical treatment according to the presented images? □ Supine position □ Other positions
4. Which type of head position would you choose for the surgical treatment according to the presented images? □ Straight “neutral” □ Straight with flexion □ Straight with extension □ Lateral rotation
5. Which approach would you choose for the surgical treatment according to the presented images? □ Supraorbital subfrontal □ Pterional □ Extended pterional
6. Which approach side would you choose for the surgical treatment according to the presented images? □ Right □ Left
7. Would you use a temporary clip for the the surgical treatment according to the presented images? □ Yes □ No
8. Which type of permanent clip would you choose for the surgical treatment according to the presented images? □ Straight/curved □ Angled □ Fenestrated

**Table 2:** Patient- and disease-related data of included patients with unruptured ACoA aneurysms operated with aneurysm clipping. One or more complications per patient were possible.

Characteristics	*n* (%), Unless otherwise Stated
age (years), mean ± SD (min-max)	54 ± 7 (30–74)
gender:	
male	12 (46)
female	14 (54)
preoperative imaging:	
CTA	26 (100)
DSA	12 (46)
MRA	20 (77)
aneurysm size:	
<11 mm	20 (77)
11–25 mm	5 (19)
>25 mm	1 (4)
morphological parameters	
mean ± SD:	
neck width	5.7 (2.3)
dome/neck ratio	1.03 (0.6)
aspect ratio	1.1 (0.6)
aneurysm angle	83.2 (14.5)
flow angle	110.8 (17.3)
aneurysm direction:	
anteriorly	12 (46)
inferiorly	9 (35)
superiorly	4 (15)
posteriorly	1 (4)
perioperative complications:	
infection	1 (4)
infarction	1 (4)
postoperative leg thrombosis	1 (4)
secondary bleeding	0 (0)
sensomotoric deficits	1 (4)
pseudomeningocele	2 (8)
discharge status:	
no new symptoms	25 (96)
new neurological symptoms	1 (4)

ACoA, anterior communicating artery; aneurysm angle, angle between the neck and the maximum height of the aneurysm; aneurysm size, the maximum perpendicular distance of the dome from the neck plane; aspect ratio, dome height/neck width; CTA, computed tomography angiography; dome/neck ratio, maximum dome width/maximum neck width; DSA, digital subtraction angiography; flow angle, angle between the maximum height of the aneurysm and the parent vessel; MRA, magnetic resonance angiography; SD, standard deviation.

**Table 3:** Assessment of the anatomical structure detection and preoperative DSA necessity * after presenting conventional CTA images (2D and screen 3D) or reconstructed 3D-VR images, evaluated using Fisher’s exact test, assuming a *p*-value <0.05 to be significant.

Image-Based Assessment, *n* (%)	CTA (*n* = 260)	3D-VR (*n* = 260)	*p*-Value
anatomical structure detection:			
appropriate	104 (40)	149 (57)	0.0001
not appropriate	156 (60)	111 (43)	(significant)
preoperative DSA:			
necessary	147 (57)	116 (45)	0.008
not necessary	113 (43)	144 (55)	(significant)

* Based on the questionnaire in Table 1. 2D, two-dimensional; 3D, three-dimensional; CTA, computed tomography angiography; DSA, digital subtraction angiography; VR, virtual reality.

**Table 4:** Recommended patient and head positioning * after presenting conventional CTA images (2D and screen 3D) or reconstructed 3D-VR images, evaluated using Fisher’s exact test, assuming a *p*-value <0.05 to be significant.

Recommendations, *n* (%)	CTA (*n* = 260)	3D-VR (*n* = 260)	*p*-Value
recommended patient positioning:			
supine position	208 (80)	226 (87)	0.38
other positions	52 (20)	34 (13)	(not significant)
recommended head positioning:			
straight “neutral”	48 (18)	28 (11)	
straight with flexion	4 (2)	8 (3)	0.005
straight with extension	24 (9)	12 (5)	(significant)
lateral rotation	184 (71)	212 (81)	

* Based on the questionnaire in Table 1. 2D, two-dimensional; 3D, three-dimensional; CTA, computed tomography angiography; VR, virtual reality.

**Table 5:** Recommended surgical approach and approach side * after presenting conventional CTA images (2D and screen 3D) or reconstructed 3D-VR images, evaluated using Fisher’s exact test, assuming a *p*-value <0.05 to be significant.

Recommendations, *n* (%)	CTA (*n* = 260)	3D-VR (*n* = 260)	*p*-Value
recommended surgical approach:			
supraorbital subfrontal	76 (29)	48 (18)	0.001
pterional	93 (36)	83 (32)	(significant)
extended pterional	91 (35)	129 (50)	
recommended approach side:			
right	135 (52)	149 (57)	0.25
left	125 (48)	111 (43)	(not significant)

* Based on the questionnaire in Table 1. 3D, three-dimensional; CTA, computed tomography angiography; VR, virtual reality.

**Table 6:** Recommended clipping strategy * after presenting conventional CTA images (2D and screen 3D) or reconstructed 3D-VR images, evaluated using Fisher’s exact test, assuming a *p*-value <0.05 to be significant.

Recommendations, *n* (%)	CTA (*n* = 260)	3D-VR (*n* = 260)	*p*-Value
temporary clipping:			
yes	74 (28)	63 (24)	0.32
no	186 (72)	197 (76)	(not significant)
type of permanent clip:			
straight/curved	175 (67)	185 (71)	0.54
angled	77 (30)	70 (27)	(not significant)
fenestrated	8 (3)	5 (2)	

* Based on the questionnaire in Table 1. 2D, two-dimensional; 3D, three-dimensional; CTA, computed tomography angiography; VR, virtual reality.
